# Ecological civilization: a revived perspective on the relationship between humanity and nature

**DOI:** 10.1093/nsr/nwab112

**Published:** 2021-06-25

**Authors:** Keping Ma, Fuwen Wei

**Affiliations:** State Key Laboratory of Vegetation and Environmental Change, Institute of Botany, Chinese Academy of Sciences, China; University of Chinese Academy of Sciences, China; CAS Key Laboratory of Animal Ecology and Conservation Biology, Institute of Zoology, Chinese Academy of Sciences, China

This is a prodigious year for introspection by humanity about our relationship with nature. The year 2021 is marked with four important global meetings: the 44th session of the World Heritage Committee of United Nations Educational, Scientific and Cultural Organization (UNESCO) scheduled in July, China; the IUCN World Conservation Congress in September, France; the 15th meeting of the Conference of the Parties (COP 15) to the Convention on Biological Diversity (CBD) in October, China; and the 26th session of the Conference of the Parties to United Nations Framework Convention on Climate Change (UNFCCC) in November, UK. All four meetings are germane to biodiversity conservation, and all will contribute to setting the future global agenda for conservation and sustainable development. In particular, CBD COP 15 will take the leading role in this regard, to review and adopt the post-2020 Global Biodiversity Framework (GBF) and associated implementation mechanisms for the 2030 mission and for the 2050 vision.

These global targets aim to bend the curve of biodiversity loss [1] by effective efforts in conservation, restoration and sustainable production and consumption. How to formulate SMART (specific, measurable, achievable, relevant and time-bound) GBF targets to support and meet the goals is the major focus, and these targets must reflect and accommodate multiple viewpoints from different parties and relevant organizations. As the host party for CBD COP 15, China is working hard in cooperation with the CBD Secretariat and relevant parties and organizations to promote the preparation of the meeting, and the associated negotiations on key documents for COP 15. The theme for the COP, ‘Ecological Civilization: building a shared future for all life on Earth’, was officially released to reflect Chinese wisdom and philosophy. China promotes the development of ecological civilization and has made remarkable progress in realizing this vision. As Xi Jinping said, ‘We need to take up our lofty responsibility for the entire human civilization, and we need to respect Nature, follow its laws and protect it. We need to find a way for man and nature to live in harmony, balance and coordinate economic development and ecological protection, and work together to build a prosperous, clean and beautiful world’ [2]. COP 15 offers an opportunity for the world to better know and understand China. In this sense, we organized a special collection of papers to showcase the critical views and research progress in biodiversity science and conservation by the academic community of China together with international colleagues.

Wei *et al*. presents the philosophical underpinnings, institutional frameworks and accomplishments of ecological civilization in China. Serving as an agent for transformative change, the concept of ecological civilization and associated actions in China provides important insights and great potential to build a shared future for all life on Earth. We hope that China's experiences can stimulate a global conversation on how best to achieve the Sustainable Development Goals (SDGs) and the goal of living in harmony with nature in a manner that respects diverse national contexts [3]. Ecological civilization is an eco-innovation, rooted in the traditional wisdom of Unity of Nature and Humanity, to harmonize the apparent contradiction between economic development and environmental protection, including biodiversity conservation. As biodiversity is a multifaceted issue intertwined with human development, integrated approaches are needed to simultaneously achieve the conservation goals and related human well-being improvements, and to seek a balance in the conceptual framework of Unity of Nature and Humanity [4]. As a critical priority, countries need to design and implement integrated national strategies to achieve the goals of the three Rio Conventions (including the UN Convention to Combat Desertification—UNCCD, UNFCCC and CBD) using spatially explicit analyses and policies. This integration can maximize co-benefits and help manage trade-offs to meet the SDGs, and maximize the retention of biodiversity and ecosystem service provision. Nature-based solutions (NBSs) provide an important framework for such integration. Countries can learn from experiences in China and elsewhere, such as Brazil's forest code to integrate nature, climate and sustainable management of land and ocean, and work to include targeted conservation and restoration of nature into their long-term low-emission development strategies [5].

Setting targets for addressing major global concerns such as biodiversity loss is an essential prerequisite for concerted global action (both inside and outside multilateral environmental agreements). However, most operational targets are socio-political choices rather than science-based decisions. Andersen *et al*. defined what ‘science-based’ means in relation to ‘science-based targets’, and to differentiate between overall science-based targets (for the world) and specific science-based targets (for individual entities). While science-based targets are necessarily ‘SMART’, the converse is not necessarily the case, because SMART targets are not necessarily underpinned by a scientific rationale [6], and for example, quantified indicators within targets may lack a scientific justification. Sound conservation policy and management relies on informed decision making with the support of advanced science and technology. China, as a megadiversity country, plays a critical role in global biodiversity conservation. China has developed national and provincial spatial-zoning plans that cover and integrate functional zones: critical ecological functions (ecological space), agricultural production (production space) and zones for industrial development and human settlements (living space) [7], which is in line with the ‘three global conditions’ [8]. By the end of 2018, China had established 11 800 protected areas, covering ∼18% of its entire land surface and 89% of species under special state protection. China supplements habitat protection with other measures such as eco-restoration and eco-compensation, which provide economic incentives to protect and restore habitats in support of wildlife conservation. However, the effective conservation and management of wildlife still presents challenges, many of which mirror challenges faced around the globe [9].

How are species and ecosystems responding and adapting to the dramatic changes in the Anthropocene? How can scientists accurately evaluate and predict the risks of local diversity loss and global extinctions and so guide the effective conservation of biodiversity? Over the past two decades, studies and publications on the issues and beyond have increased dramatically in quantity and quality. The number of papers on China's biodiversity published in international journals, as an example, has grown from a few dozen in 2000 to over 1700 in 2019. A comprehensive review of the biodiversity science output led by Chinese institutions and published in interdisciplinary international journals over the last two decades was carried out across three broad categories: inventory and monitoring; processes and mechanisms; and threats and responses. Priorities for future research were also proposed [10,11]. The systematic review was carried out in both a theoretical and practical sense. The achievements in biodiversity science by Chinese scientists and their collaborators are highly valued by international colleagues who also advocated insightful future priorities for further development [12–14]. Given the rapid evolution of biodiversity research and conservation within China and increasing international leadership, China is well positioned to become a global leader in the field of biodiversity science in the near future, helping other countries around the world on the path towards ecological civilization [10].
